# Diaqua-μ_3_-oxido-hexa­kis(μ_2_-trichloro­acetato-κ^2^ *O*:*O*′)(trichloro­acetato-κ*O*)trichromium(III) acetonitrile tris­olvate

**DOI:** 10.1107/S1600536808025798

**Published:** 2008-08-16

**Authors:** B. B. Mougang D. Soume, Rosiyah Yahya, Seng Neon Gan, Seik Weng Ng

**Affiliations:** a Department of Chemistry, University of Malaya, 50603 Kuala Lumpur, Malaysia

## Abstract

In the crystal structure of the title compound, [Cr_3_(C_2_Cl_3_O_2_)_7_O(H_2_O)_2_]·3CH_3_CN, the trinuclear [Cr_3_O(H_2_O)_2_(Cl_3_CCO_2_)_7_] mol­ecule has an oxide O atom that is connected to one monodentate trichloro­acetate-coordinated and two water-coordinated Cr^III^ atoms, the three metal atoms forming the points of an equilateral triangle. Each of the six remaining carboxyl­ate groups bridges a Cr–O–Cr fragment. The cluster inter­acts with the three solvent mol­ecules through water–acetonitrile O—H⋯N hydrogen bonds. Adjacent clusters are linked by a water–carboxylate O—H⋯O hydrogen bond to give a helical chain. One of the CCl_3_ groups was found to be disordered over two positions, with the major component having a site-occupancy factor of 0.64 (1).

## Related literature

Oxo-centred chromium(III) chloro­acetates form an efficient class of Zigler–Natta catalysts for the polymerization of olefins; see: Gan *et al.* (2000 ▶).
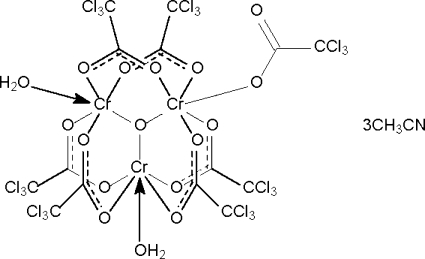


## Experimental

### 

#### Crystal data

[Cr_3_(C_2_Cl_3_O_2_)_7_O(H_2_O)_2_]·3C_2_H_3_N
*M* *_r_* = 1467.78
Monoclinic, 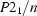

*a* = 11.6307 (6) Å
*b* = 19.481 (1) Å
*c* = 22.949 (1) Å
β = 95.355 (1)°
*V* = 5177.0 (5) Å^3^
*Z* = 4
Mo *K*α radiation
μ = 1.76 mm^−1^
*T* = 100 (2) K
0.25 × 0.20 × 0.15 mm

#### Data collection

Bruker SMART APEX diffractometer
Absorption correction: multi-scan (*SADABS*; Sheldrick, 1996 ▶) *T*_min_ = 0.667, *T*_max_ = 0.778
29504 measured reflections
11718 independent reflections
9590 reflections with *I* > 2σ(*I*)
*R*_int_ = 0.029

#### Refinement

*R*[*F* ^2^ > 2σ(*F* ^2^)] = 0.041
*wR*(*F* ^2^) = 0.118
*S* = 1.04
11718 reflections
624 parameters
72 restraints
H atoms treated by a mixture of independent and constrained refinement
Δρ_max_ = 1.42 e Å^−3^
Δρ_min_ = −0.74 e Å^−3^

### 

Data collection: *APEX2* (Bruker, 2007 ▶); cell refinement: *SAINT* (Bruker, 2007 ▶); data reduction: *SAINT*; program(s) used to solve structure: *SHELXS97* (Sheldrick, 2008 ▶); program(s) used to refine structure: *SHELXL97* (Sheldrick, 2008 ▶); molecular graphics: *X-SEED* (Barbour, 2001 ▶); software used to prepare material for publication: *publCIF* (Westrip, 2008 ▶).

## Supplementary Material

Crystal structure: contains datablocks global, I. DOI: 10.1107/S1600536808025798/tk2293sup1.cif

Structure factors: contains datablocks I. DOI: 10.1107/S1600536808025798/tk2293Isup2.hkl

Additional supplementary materials:  crystallographic information; 3D view; checkCIF report

## Figures and Tables

**Table 1:** Hydrogen-bond geometry (Å, °)

*D*—H⋯*A*	*D*—H	H⋯*A*	*D*⋯*A*	*D*—H⋯*A*
O1*w*—H1*w*1⋯N1	0.84 (1)	1.97 (2)	2.791 (4)	166 (5)
O1*w*—H1*w*2⋯N2	0.85 (1)	1.97 (1)	2.810 (5)	173 (4)
O2*w*—H2*w*1⋯N3	0.85 (1)	1.88 (1)	2.719 (5)	171 (4)
O2*w*—H2*w*2⋯O2^i^	0.83 (1)	1.81 (1)	2.613 (3)	165 (4)

Symmetry code: (i) 
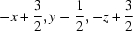
.

**Fig. 1.**
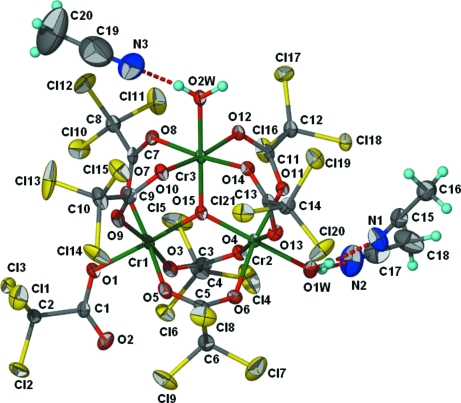
Thermal ellipsoid plot (Barbour, 2001) plot of [Cr3O(H2O)2(Cl3CCO2)7].3CH3CN at the 70% probability level. Hydrogen atoms are drawn as spheres of arbitrary radius. For clarity, the minor disorder component is not shown. Hydrogen bonds are denoted by dashed lines.

## supplementary crystallographic information

### Comment

Oxo-centered chromium(III) chloroacetates form an efficient class of Zigler–Natta catalysts for the polymerization of olefins (Gan *et al.*, 2000). The title trichloroacetate oxo-cluster crystallizes with acetonitrile (Scheme I, Fig. 1). In the crystal structure, the oxo-O atom is connected to one monodentate trichloroacetate-coordinated and two water-coordinated chromium(III) atoms, the three metal atoms forming the points of an equilateral triangle. Each of the six remaining carboxylate groups chelates a Cr–O–Cr fragment. The cluster interacts with the three solvate molecules through hydrogen bonds; hydrogen bonds involving the water molecule as a donor give rise to a helical chain that runs along the *b*-axis.

### Experimental

Chromium(III) chloride hexahydrate (10 g) was refluxed with trichloroacetic acid in a molar ratio of 1:6 for 6 h. The solution was filtered hot; the cooled solution yielded a green product that was washed with chloroform (90% yield). Analysis found: Cr, 11.13, C, 11.98, H, 0.72; Cr_3_Cl_21_C_14_O_20_H_10_ requires: Cr, 11.15, C, 12.02, H, 0.72. Dark-green crystals of the acetonitrile solvate were obtained by recrystallization from an acetonitrile solution.

### Refinement

One of the seven trichloroacetate groups was found to be disordered over two sites. The six C—Cl distances were restrained to within 0.01 Å of each other, as were the Cl···Cl distances. The anisotropic displacement parameters of the disordered Cl atoms were restrained to be nearly isotropic. The disorder refined to a 0.636 (12):0.364 (12) site occupancy ratio. The water hydrogen atoms were located in a difference Fourier map, and were refined with a distance restraint of O—H 0.84 (1) Å; their temperature factors were freely refined. The methyl-H atoms were generated geometrically (C—H = 0.98 Å), and were included in the refinement in the riding model approximation with *U*_iso_(H) = 1.5*U*_eq_(C). The final difference Fourier map had a large peak in the vicinity of the disordered Cl atoms, but was otherwise featureless.

### Crystal data

**Table:**

[Cr_3_(C_2_Cl_3_O_2_)_7_O(H_2_O)_2_]·3C_2_H_3_N	*F*_000_ = 2876
*M**_r_* = 1467.78	*D*_x_ = 1.883 Mg m^−^^3^
Monoclinic, *P*2_1_/*n*	Mo *K*α radiation λ = 0.71073 Å
Hall symbol: -P 2yn	Cell parameters from 9535 reflections
*a* = 11.6307 (6) Å	θ = 2.2–28.2º
*b* = 19.481 (1) Å	µ = 1.76 mm^−^^1^
*c* = 22.949 (1) Å	*T* = 100 (2) K
β = 95.355 (1)º	Block, green
*V* = 5177.0 (5) Å^3^	0.25 × 0.20 × 0.15 mm
*Z* = 4	

### Data collection

**Table:**

Bruker SMART APEX diffractometer	9590 reflections with *I* > 2σ(*I*)
Radiation source: fine-focus sealed tube	*R*_int_ = 0.029
Monochromator: graphite	θ_max_ = 27.5º
ω scans	θ_min_ = 1.4º
Absorption correction: multi-scan(SADABS; Sheldrick, 1996)	*h* = −15→11
*T*_min_ = 0.667, *T*_max_ = 0.778	*k* = −25→25
29504 measured reflections	*l* = −29→29
11718 independent reflections	

### Refinement

**Table:**

Refinement on *F*^2^	Secondary atom site location: difference Fourier map
Least-squares matrix: full	Hydrogen site location: inferred from neighbouring sites
*R*[*F*^2^ > 2σ(*F*^2^)] = 0.041	H atoms treated by a mixture of independent and constrained refinement
*wR*(*F*^2^) = 0.118	*w* = 1/[σ^2^(*F*_o_^2^) + (0.0626*P*)^2^ + 7.2035*P*] where *P* = (*F*_o_^2^ + 2*F*_c_^2^)/3
*S* = 1.04	(Δ/σ)_max_ = 0.001
11718 reflections	Δρ_max_ = 1.42 e Å^−^^3^
624 parameters	Δρ_min_ = −0.74 e Å^−^^3^
72 restraints	Extinction correction: none
Primary atom site location: structure-invariant direct methods	

### Fractional atomic coordinates and isotropic or equivalent isotropic displacement parameters (Å^2^)

**Table:**

	*x*	*y*	*z*	*U*_iso_*/*U*_eq_	Occ. (<1)
Cr1	0.74255 (4)	0.76778 (3)	0.70072 (2)	0.01404 (12)	
Cr2	0.68641 (4)	0.73747 (3)	0.83719 (2)	0.01277 (11)	
Cr3	0.72390 (4)	0.60693 (3)	0.74830 (2)	0.01179 (11)	
Cl1	0.96872 (8)	0.85657 (6)	0.56393 (4)	0.0333 (2)	
Cl2	0.89615 (9)	0.99687 (5)	0.57400 (4)	0.0345 (2)	
Cl3	0.73830 (8)	0.89677 (4)	0.52068 (3)	0.02158 (18)	
Cl4	0.36046 (10)	0.88419 (6)	0.80350 (5)	0.0431 (3)	
Cl5	0.35756 (9)	0.81589 (5)	0.69151 (5)	0.0430 (3)	
Cl6	0.46821 (8)	0.94801 (4)	0.70989 (4)	0.02438 (19)	
Cl7	0.98178 (10)	0.88074 (7)	0.90381 (5)	0.0451 (3)	
Cl8	1.09295 (9)	0.78569 (5)	0.83228 (5)	0.0346 (2)	
Cl9	1.03479 (9)	0.92225 (5)	0.78947 (5)	0.0356 (2)	
Cl10	0.44418 (9)	0.70978 (5)	0.55064 (4)	0.0322 (2)	
Cl11	0.38086 (9)	0.61375 (7)	0.63683 (5)	0.0399 (3)	
Cl12	0.53908 (9)	0.57331 (5)	0.55303 (4)	0.0336 (2)	
Cl13	0.9998 (4)	0.6385 (3)	0.58065 (14)	0.0556 (10)	0.636 (12)
Cl14	1.1108 (2)	0.71821 (13)	0.67552 (16)	0.0352 (8)	0.636 (12)
Cl15	1.0981 (8)	0.5711 (3)	0.6842 (4)	0.0316 (13)	0.636 (12)
Cl3'	0.9729 (10)	0.6101 (9)	0.5847 (3)	0.105 (5)	0.364 (12)
Cl4'	1.1068 (6)	0.7141 (3)	0.6463 (8)	0.103 (3)	0.364 (12)
Cl5'	1.1046 (14)	0.5774 (5)	0.6926 (7)	0.0252 (17)	0.364 (12)
Cl16	0.31914 (8)	0.66992 (5)	0.78820 (4)	0.0287 (2)	
Cl17	0.35454 (8)	0.52405 (5)	0.79999 (4)	0.0292 (2)	
Cl18	0.37880 (7)	0.61005 (4)	0.90215 (3)	0.01931 (17)	
Cl19	0.93022 (8)	0.51554 (5)	0.92623 (4)	0.0272 (2)	
Cl20	0.99064 (8)	0.65247 (6)	0.96451 (4)	0.0344 (2)	
Cl21	1.07222 (7)	0.60054 (5)	0.85829 (4)	0.02607 (19)	
O1	0.7710 (2)	0.82999 (12)	0.63619 (10)	0.0187 (5)	
O2	0.8135 (3)	0.93428 (14)	0.67484 (11)	0.0281 (6)	
O3	0.6252 (2)	0.83313 (12)	0.72363 (10)	0.0179 (5)	
O4	0.55621 (19)	0.79526 (12)	0.80588 (10)	0.0163 (5)	
O5	0.8720 (2)	0.81086 (13)	0.74973 (10)	0.0197 (5)	
O6	0.8001 (2)	0.81248 (12)	0.83713 (10)	0.0184 (5)	
O7	0.6175 (2)	0.72533 (12)	0.64643 (10)	0.0178 (5)	
O8	0.6429 (2)	0.61373 (12)	0.66921 (9)	0.0160 (5)	
O9	0.8600 (2)	0.71060 (12)	0.66684 (10)	0.0184 (5)	
O10	0.8769 (2)	0.61126 (12)	0.71653 (10)	0.0168 (5)	
O11	0.57304 (19)	0.66600 (12)	0.85012 (10)	0.0155 (5)	
O12	0.56962 (19)	0.58956 (12)	0.77652 (9)	0.0147 (5)	
O13	0.81379 (19)	0.68469 (12)	0.87790 (10)	0.0169 (5)	
O14	0.81080 (19)	0.58767 (12)	0.82463 (9)	0.0151 (5)	
O15	0.71611 (19)	0.70351 (11)	0.76204 (9)	0.0136 (4)	
O1W	0.6544 (2)	0.77209 (13)	0.91690 (10)	0.0190 (5)	
H1W1	0.684 (3)	0.747 (2)	0.9440 (15)	0.059 (17)*	
H1W2	0.5872 (17)	0.783 (2)	0.9250 (17)	0.034 (13)*	
O2W	0.7303 (2)	0.50636 (12)	0.73377 (10)	0.0171 (5)	
H2W1	0.759 (4)	0.4871 (18)	0.7053 (11)	0.032 (12)*	
H2W2	0.726 (4)	0.4790 (17)	0.7610 (12)	0.050 (15)*	
N1	0.7157 (3)	0.67991 (19)	1.00764 (16)	0.0338 (8)	
N2	0.4284 (4)	0.7968 (3)	0.9455 (2)	0.0556 (12)	
N3	0.8447 (5)	0.4523 (3)	0.6464 (2)	0.0685 (15)	
C1	0.8074 (3)	0.89117 (17)	0.63661 (14)	0.0157 (6)	
C2	0.8515 (3)	0.91082 (17)	0.57647 (14)	0.0172 (7)	
C3	0.5520 (3)	0.82935 (16)	0.75960 (14)	0.0158 (6)	
C4	0.4390 (3)	0.86978 (19)	0.74313 (17)	0.0229 (7)	
C5	0.8757 (3)	0.82309 (17)	0.80318 (15)	0.0173 (7)	
C6	0.9912 (3)	0.85391 (19)	0.83110 (15)	0.0215 (7)	
C7	0.5957 (3)	0.66336 (18)	0.64181 (13)	0.0156 (6)	
C8	0.4943 (3)	0.64175 (18)	0.59620 (15)	0.0196 (7)	
C9	0.9091 (3)	0.65627 (17)	0.68302 (14)	0.0161 (6)	
C10	1.0241 (3)	0.64354 (16)	0.65623 (14)	0.0254 (8)	
C11	0.5281 (3)	0.62049 (17)	0.81711 (14)	0.0142 (6)	
C12	0.3999 (3)	0.60421 (18)	0.82725 (14)	0.0177 (7)	
C13	0.8484 (3)	0.62681 (17)	0.86503 (14)	0.0144 (6)	
C14	0.9565 (3)	0.59973 (18)	0.90309 (15)	0.0187 (7)	
C15	0.7356 (3)	0.6309 (2)	1.03291 (16)	0.0255 (8)	
C16	0.7599 (4)	0.5680 (2)	1.06581 (18)	0.0342 (9)	
H16A	0.8074	0.5785	1.1023	0.051*	
H16B	0.8016	0.5360	1.0424	0.051*	
H16C	0.6872	0.5470	1.0750	0.051*	
C17	0.3472 (4)	0.7913 (2)	0.9685 (2)	0.0423 (11)	
C18	0.2451 (4)	0.7862 (3)	0.9985 (3)	0.0504 (13)	
H18A	0.2630	0.7620	1.0356	0.076*	
H18B	0.1858	0.7609	0.9742	0.076*	
H18C	0.2167	0.8324	1.0062	0.076*	
C19	0.8283 (8)	0.4449 (4)	0.5933 (4)	0.091 (2)	
C20	0.8044 (9)	0.4336 (6)	0.5267 (3)	0.126 (4)	
H20A	0.8704	0.4102	0.5119	0.189*	
H20B	0.7925	0.4781	0.5071	0.189*	
H20C	0.7350	0.4053	0.5187	0.189*	

### Atomic displacement parameters (Å^2^)

**Table:**

	*U*^11^	*U*^22^	*U*^33^	*U*^12^	*U*^13^	*U*^23^
Cr1	0.0128 (3)	0.0158 (3)	0.0139 (2)	0.0004 (2)	0.00299 (19)	0.00348 (19)
Cr2	0.0104 (2)	0.0147 (3)	0.0134 (2)	−0.00009 (19)	0.00208 (18)	0.00052 (19)
Cr3	0.0095 (2)	0.0149 (3)	0.0112 (2)	0.00076 (19)	0.00208 (18)	0.00201 (18)
Cl1	0.0189 (5)	0.0495 (6)	0.0329 (5)	0.0045 (4)	0.0097 (4)	−0.0016 (4)
Cl2	0.0441 (6)	0.0272 (5)	0.0306 (5)	−0.0214 (4)	−0.0047 (4)	0.0062 (4)
Cl3	0.0244 (4)	0.0247 (4)	0.0147 (4)	−0.0056 (3)	−0.0031 (3)	−0.0002 (3)
Cl4	0.0320 (6)	0.0450 (6)	0.0567 (7)	0.0210 (5)	0.0277 (5)	0.0231 (5)
Cl5	0.0313 (6)	0.0279 (5)	0.0645 (7)	−0.0076 (4)	−0.0240 (5)	0.0102 (5)
Cl6	0.0246 (4)	0.0168 (4)	0.0320 (5)	0.0038 (3)	0.0037 (3)	0.0075 (3)
Cl7	0.0338 (6)	0.0700 (8)	0.0317 (5)	−0.0181 (5)	0.0043 (4)	−0.0204 (5)
Cl8	0.0231 (5)	0.0291 (5)	0.0492 (6)	0.0034 (4)	−0.0098 (4)	0.0042 (4)
Cl9	0.0253 (5)	0.0303 (5)	0.0495 (6)	−0.0113 (4)	−0.0057 (4)	0.0172 (4)
Cl10	0.0359 (5)	0.0282 (5)	0.0291 (5)	0.0015 (4)	−0.0152 (4)	0.0076 (4)
Cl11	0.0192 (5)	0.0669 (8)	0.0334 (5)	−0.0113 (5)	0.0016 (4)	0.0109 (5)
Cl12	0.0399 (6)	0.0328 (5)	0.0258 (5)	0.0067 (4)	−0.0081 (4)	−0.0095 (4)
Cl13	0.0395 (16)	0.112 (3)	0.0170 (11)	0.0297 (15)	0.0124 (9)	0.0041 (11)
Cl14	0.0124 (10)	0.0279 (10)	0.0660 (18)	−0.0058 (7)	0.0066 (10)	0.0156 (10)
Cl15	0.0214 (19)	0.0290 (15)	0.047 (3)	0.0084 (12)	0.0165 (17)	0.0106 (15)
Cl3'	0.076 (6)	0.221 (13)	0.020 (2)	0.086 (7)	0.014 (3)	−0.001 (5)
Cl4'	0.050 (3)	0.058 (3)	0.214 (9)	0.025 (2)	0.084 (5)	0.081 (4)
Cl5'	0.017 (2)	0.030 (3)	0.029 (3)	0.012 (2)	0.0058 (17)	0.008 (2)
Cl16	0.0158 (4)	0.0420 (6)	0.0283 (5)	0.0072 (4)	0.0009 (3)	0.0121 (4)
Cl17	0.0231 (5)	0.0326 (5)	0.0336 (5)	−0.0133 (4)	0.0119 (4)	−0.0121 (4)
Cl18	0.0165 (4)	0.0251 (4)	0.0174 (4)	−0.0001 (3)	0.0070 (3)	0.0011 (3)
Cl19	0.0242 (5)	0.0303 (5)	0.0259 (4)	0.0024 (4)	−0.0043 (3)	0.0115 (4)
Cl20	0.0232 (5)	0.0452 (6)	0.0318 (5)	0.0122 (4)	−0.0136 (4)	−0.0189 (4)
Cl21	0.0117 (4)	0.0285 (5)	0.0387 (5)	0.0009 (3)	0.0060 (3)	−0.0024 (4)
O1	0.0216 (13)	0.0186 (12)	0.0162 (11)	−0.0034 (10)	0.0031 (9)	0.0034 (9)
O2	0.0416 (17)	0.0240 (14)	0.0190 (13)	0.0025 (12)	0.0051 (11)	−0.0055 (10)
O3	0.0166 (12)	0.0177 (12)	0.0201 (12)	0.0016 (9)	0.0046 (9)	0.0038 (9)
O4	0.0140 (12)	0.0170 (12)	0.0182 (11)	0.0018 (9)	0.0027 (9)	0.0030 (9)
O5	0.0162 (12)	0.0244 (13)	0.0186 (12)	−0.0050 (10)	0.0022 (9)	0.0053 (10)
O6	0.0168 (12)	0.0189 (12)	0.0201 (12)	−0.0048 (9)	0.0053 (9)	−0.0020 (9)
O7	0.0180 (12)	0.0196 (12)	0.0156 (11)	0.0002 (9)	0.0007 (9)	0.0041 (9)
O8	0.0174 (12)	0.0169 (12)	0.0135 (11)	0.0015 (9)	0.0003 (9)	0.0011 (9)
O9	0.0163 (12)	0.0211 (12)	0.0186 (12)	0.0029 (10)	0.0064 (9)	0.0052 (9)
O10	0.0132 (11)	0.0190 (12)	0.0188 (11)	0.0022 (9)	0.0049 (9)	0.0041 (9)
O11	0.0138 (11)	0.0172 (12)	0.0158 (11)	−0.0017 (9)	0.0039 (9)	0.0000 (9)
O12	0.0114 (11)	0.0182 (12)	0.0148 (11)	−0.0002 (9)	0.0032 (8)	0.0013 (9)
O13	0.0129 (11)	0.0203 (12)	0.0172 (11)	0.0032 (9)	−0.0001 (9)	−0.0012 (9)
O14	0.0132 (11)	0.0164 (11)	0.0154 (11)	0.0001 (9)	−0.0001 (9)	0.0023 (9)
O15	0.0124 (11)	0.0150 (11)	0.0138 (11)	0.0027 (9)	0.0035 (8)	0.0023 (8)
O1W	0.0174 (13)	0.0230 (13)	0.0173 (12)	0.0018 (10)	0.0050 (10)	−0.0007 (10)
O2W	0.0216 (13)	0.0163 (12)	0.0140 (11)	0.0016 (10)	0.0046 (9)	0.0022 (9)
N1	0.0257 (18)	0.038 (2)	0.038 (2)	0.0025 (15)	0.0060 (15)	0.0135 (16)
N2	0.047 (3)	0.076 (3)	0.046 (3)	0.011 (2)	0.013 (2)	0.000 (2)
N3	0.089 (4)	0.057 (3)	0.067 (3)	−0.005 (3)	0.046 (3)	−0.022 (3)
C1	0.0133 (16)	0.0204 (17)	0.0135 (15)	0.0014 (13)	0.0016 (12)	0.0020 (12)
C2	0.0181 (17)	0.0179 (16)	0.0154 (15)	−0.0046 (13)	0.0001 (13)	0.0015 (12)
C3	0.0132 (16)	0.0124 (15)	0.0217 (16)	0.0001 (12)	0.0014 (12)	0.0016 (12)
C4	0.0183 (18)	0.0192 (18)	0.031 (2)	0.0029 (14)	0.0032 (14)	0.0066 (14)
C5	0.0150 (16)	0.0137 (15)	0.0234 (17)	−0.0021 (12)	0.0023 (13)	0.0028 (13)
C6	0.0162 (17)	0.0268 (19)	0.0212 (17)	−0.0045 (14)	0.0004 (13)	0.0014 (14)
C7	0.0144 (16)	0.0225 (17)	0.0106 (14)	0.0022 (13)	0.0040 (12)	0.0026 (12)
C8	0.0195 (17)	0.0212 (17)	0.0177 (16)	0.0015 (14)	0.0005 (13)	0.0033 (13)
C9	0.0112 (15)	0.0218 (17)	0.0152 (15)	0.0011 (13)	0.0013 (12)	−0.0005 (12)
C10	0.0178 (18)	0.031 (2)	0.0286 (19)	0.0065 (15)	0.0093 (14)	0.0119 (15)
C11	0.0100 (15)	0.0182 (16)	0.0145 (15)	0.0012 (12)	0.0022 (11)	0.0065 (12)
C12	0.0139 (16)	0.0223 (17)	0.0175 (16)	0.0006 (13)	0.0049 (12)	0.0013 (13)
C13	0.0083 (15)	0.0206 (17)	0.0146 (15)	−0.0007 (12)	0.0022 (11)	0.0023 (12)
C14	0.0129 (16)	0.0236 (18)	0.0191 (16)	0.0021 (13)	−0.0012 (13)	−0.0023 (13)
C15	0.0207 (19)	0.032 (2)	0.0237 (18)	−0.0009 (16)	0.0027 (14)	0.0001 (16)
C16	0.046 (3)	0.028 (2)	0.028 (2)	0.0033 (18)	−0.0004 (18)	0.0012 (16)
C17	0.036 (3)	0.039 (3)	0.052 (3)	0.008 (2)	0.005 (2)	−0.004 (2)
C18	0.035 (3)	0.036 (3)	0.082 (4)	0.002 (2)	0.018 (3)	0.008 (2)
C19	0.110 (6)	0.059 (4)	0.112 (7)	0.002 (4)	0.052 (5)	−0.005 (4)
C20	0.133 (9)	0.177 (10)	0.073 (6)	0.024 (7)	0.036 (5)	−0.032 (6)

### Geometric parameters (Å, °)

**Table:**

Cr1—O15	1.930 (2)	Cl21—C14	1.768 (4)
Cr1—O1	1.965 (2)	O1—C1	1.264 (4)
Cr1—O3	1.973 (2)	O2—C1	1.212 (4)
Cr1—O9	1.978 (2)	O3—C3	1.242 (4)
Cr1—O5	1.980 (2)	O4—C3	1.250 (4)
Cr1—O7	2.003 (2)	O5—C5	1.246 (4)
Cr2—O15	1.909 (2)	O6—C5	1.246 (4)
Cr2—O11	1.959 (2)	O7—C7	1.236 (4)
Cr2—O13	1.966 (2)	O8—C7	1.252 (4)
Cr2—O4	1.968 (2)	O9—C9	1.243 (4)
Cr2—O6	1.971 (2)	O10—C9	1.247 (4)
Cr2—O1W	2.017 (2)	O11—C11	1.249 (4)
Cr3—O15	1.911 (2)	O12—C11	1.244 (4)
Cr3—O8	1.970 (2)	O13—C13	1.242 (4)
Cr3—O14	1.975 (2)	O14—C13	1.248 (4)
Cr3—O10	1.986 (2)	O1W—H1W1	0.839 (10)
Cr3—O2W	1.990 (2)	O1W—H1W2	0.849 (10)
Cr3—O12	1.993 (2)	O2W—H2W1	0.850 (10)
Cl1—C2	1.769 (4)	O2W—H2W2	0.826 (10)
Cl2—C2	1.757 (3)	N1—C15	1.131 (5)
Cl3—C2	1.770 (3)	N2—C17	1.130 (6)
Cl4—C4	1.752 (4)	N3—C19	1.225 (10)
Cl5—C4	1.787 (4)	C1—C2	1.564 (4)
Cl6—C4	1.752 (4)	C3—C4	1.549 (5)
Cl7—C6	1.762 (4)	C5—C6	1.554 (5)
Cl8—C6	1.778 (4)	C7—C8	1.560 (5)
Cl9—C6	1.741 (4)	C9—C10	1.543 (5)
Cl10—C8	1.754 (3)	C11—C12	1.562 (4)
Cl11—C8	1.772 (4)	C13—C14	1.555 (4)
Cl12—C8	1.768 (4)	C15—C16	1.453 (5)
Cl13—C10	1.734 (4)	C16—H16A	0.9800
Cl14—C10	1.802 (4)	C16—H16B	0.9800
Cl15—C10	1.744 (5)	C16—H16C	0.9800
Cl3'—C10	1.813 (6)	C17—C18	1.429 (7)
Cl4'—C10	1.705 (5)	C18—H18A	0.9800
Cl5'—C10	1.757 (6)	C18—H18B	0.9800
Cl16—C12	1.780 (4)	C18—H18C	0.9800
Cl17—C12	1.745 (4)	C19—C20	1.544 (11)
Cl18—C12	1.762 (3)	C20—H20A	0.9800
Cl19—C14	1.759 (4)	C20—H20B	0.9800
Cl20—C14	1.759 (3)	C20—H20C	0.9800
			
O15—Cr1—O1	177.60 (10)	C3—C4—Cl6	110.7 (2)
O15—Cr1—O3	93.62 (9)	Cl4—C4—Cl6	110.0 (2)
O1—Cr1—O3	88.40 (10)	C3—C4—Cl5	104.4 (2)
O15—Cr1—O9	94.83 (9)	Cl4—C4—Cl5	109.6 (2)
O1—Cr1—O9	83.11 (10)	Cl6—C4—Cl5	109.6 (2)
O3—Cr1—O9	171.35 (10)	O6—C5—O5	128.5 (3)
O15—Cr1—O5	91.45 (10)	O6—C5—C6	116.1 (3)
O1—Cr1—O5	89.66 (10)	O5—C5—C6	115.4 (3)
O3—Cr1—O5	94.78 (10)	C5—C6—Cl9	110.7 (2)
O9—Cr1—O5	86.86 (10)	C5—C6—Cl7	112.2 (2)
O15—Cr1—O7	91.71 (9)	Cl9—C6—Cl7	109.9 (2)
O1—Cr1—O7	87.10 (10)	C5—C6—Cl8	105.4 (2)
O3—Cr1—O7	87.27 (10)	Cl9—C6—Cl8	110.6 (2)
O9—Cr1—O7	90.63 (10)	Cl7—C6—Cl8	107.91 (19)
O5—Cr1—O7	176.12 (10)	O7—C7—O8	129.4 (3)
O15—Cr2—O11	94.11 (10)	O7—C7—C8	117.2 (3)
O15—Cr2—O13	93.23 (9)	O8—C7—C8	113.4 (3)
O11—Cr2—O13	92.54 (10)	C7—C8—Cl10	112.6 (2)
O15—Cr2—O4	93.81 (9)	C7—C8—Cl12	109.6 (2)
O11—Cr2—O4	87.48 (10)	Cl10—C8—Cl12	109.56 (19)
O13—Cr2—O4	172.95 (10)	C7—C8—Cl11	106.5 (2)
O15—Cr2—O6	94.50 (10)	Cl10—C8—Cl11	108.88 (19)
O11—Cr2—O6	171.16 (10)	Cl12—C8—Cl11	109.6 (2)
O13—Cr2—O6	84.97 (10)	O9—C9—O10	128.8 (3)
O4—Cr2—O6	93.95 (10)	O9—C9—C10	114.1 (3)
O15—Cr2—O1W	179.24 (10)	C9—C10—Cl4'	116.4 (4)
O11—Cr2—O1W	85.21 (10)	C9—C10—Cl13	110.0 (3)
O13—Cr2—O1W	86.47 (10)	C9—C10—Cl15	113.5 (4)
O4—Cr2—O1W	86.50 (10)	Cl4'—C10—Cl15	115.9 (5)
O6—Cr2—O1W	86.17 (10)	Cl13—C10—Cl15	110.6 (4)
O15—Cr3—O8	93.43 (9)	C9—C10—Cl5'	111.8 (7)
O15—Cr3—O14	93.95 (9)	Cl4'—C10—Cl5'	111.8 (5)
O8—Cr3—O14	172.40 (10)	Cl13—C10—Cl5'	117.6 (7)
O15—Cr3—O10	94.39 (10)	C9—C10—Cl14	105.0 (2)
O8—Cr3—O10	91.54 (10)	Cl13—C10—Cl14	109.1 (2)
O14—Cr3—O10	86.09 (10)	Cl15—C10—Cl14	108.5 (3)
O15—Cr3—O2W	179.43 (10)	Cl5'—C10—Cl14	102.3 (5)
O8—Cr3—O2W	86.22 (10)	C9—C10—Cl3'	101.2 (4)
O14—Cr3—O2W	86.41 (10)	Cl4'—C10—Cl3'	108.1 (4)
O10—Cr3—O2W	86.07 (10)	Cl5'—C10—Cl3'	106.4 (5)
O15—Cr3—O12	93.18 (9)	Cl14—C10—Cl3'	129.9 (5)
O8—Cr3—O12	86.73 (9)	O12—C11—O11	128.9 (3)
O14—Cr3—O12	94.66 (9)	O12—C11—C12	117.1 (3)
O10—Cr3—O12	172.32 (10)	O11—C11—C12	113.9 (3)
O2W—Cr3—O12	86.35 (10)	C11—C12—Cl17	112.9 (2)
C1—O1—Cr1	130.9 (2)	C11—C12—Cl18	110.7 (2)
C3—O3—Cr1	132.3 (2)	Cl17—C12—Cl18	110.02 (18)
C3—O4—Cr2	125.6 (2)	C11—C12—Cl16	104.2 (2)
C5—O5—Cr1	126.5 (2)	Cl17—C12—Cl16	109.59 (19)
C5—O6—Cr2	129.5 (2)	Cl18—C12—Cl16	109.25 (18)
C7—O7—Cr1	126.0 (2)	O13—C13—O14	129.3 (3)
C7—O8—Cr3	131.6 (2)	O13—C13—C14	115.9 (3)
C9—O9—Cr1	132.6 (2)	O14—C13—C14	114.8 (3)
C9—O10—Cr3	126.0 (2)	C13—C14—Cl19	109.4 (2)
C11—O11—Cr2	131.5 (2)	C13—C14—Cl20	111.2 (2)
C11—O12—Cr3	125.8 (2)	Cl19—C14—Cl20	109.58 (19)
C13—O13—Cr2	127.4 (2)	C13—C14—Cl21	106.9 (2)
C13—O14—Cr3	131.1 (2)	Cl19—C14—Cl21	110.30 (19)
Cr2—O15—Cr3	120.38 (11)	Cl20—C14—Cl21	109.49 (19)
Cr2—O15—Cr1	119.25 (12)	N1—C15—C16	179.3 (5)
Cr3—O15—Cr1	120.35 (11)	C15—C16—H16A	109.5
Cr2—O1W—H1W1	112 (3)	C15—C16—H16B	109.5
Cr2—O1W—H1W2	122 (3)	H16A—C16—H16B	109.5
H1W1—O1W—H1W2	108.1 (17)	C15—C16—H16C	109.5
Cr3—O2W—H2W1	126 (3)	H16A—C16—H16C	109.5
Cr3—O2W—H2W2	120 (3)	H16B—C16—H16C	109.5
H2W1—O2W—H2W2	110.6 (18)	N2—C17—C18	178.2 (6)
O2—C1—O1	131.0 (3)	C17—C18—H18A	109.5
O2—C1—C2	117.9 (3)	C17—C18—H18B	109.5
O1—C1—C2	111.2 (3)	H18A—C18—H18B	109.5
C1—C2—Cl2	112.6 (2)	C17—C18—H18C	109.5
C1—C2—Cl3	108.5 (2)	H18A—C18—H18C	109.5
Cl2—C2—Cl3	109.03 (18)	H18B—C18—H18C	109.5
C1—C2—Cl1	108.6 (2)	N3—C19—C20	178.0 (9)
Cl2—C2—Cl1	109.29 (19)	C19—C20—H20A	109.5
Cl3—C2—Cl1	108.81 (18)	C19—C20—H20B	109.5
O3—C3—O4	128.4 (3)	H20A—C20—H20B	109.5
O3—C3—C4	115.3 (3)	C19—C20—H20C	109.5
O4—C3—C4	116.3 (3)	H20A—C20—H20C	109.5
C3—C4—Cl4	112.4 (2)	H20B—C20—H20C	109.5
			
O3—Cr1—O1—C1	62.2 (3)	O7—Cr1—O15—Cr3	56.08 (14)
O9—Cr1—O1—C1	−119.5 (3)	Cr1—O1—C1—O2	−17.1 (6)
O5—Cr1—O1—C1	−32.6 (3)	Cr1—O1—C1—C2	162.3 (2)
O7—Cr1—O1—C1	149.5 (3)	O2—C1—C2—Cl2	−3.5 (4)
O15—Cr1—O3—C3	−7.9 (3)	O1—C1—C2—Cl2	177.0 (2)
O1—Cr1—O3—C3	170.8 (3)	O2—C1—C2—Cl3	−124.3 (3)
O5—Cr1—O3—C3	−99.7 (3)	O1—C1—C2—Cl3	56.2 (3)
O7—Cr1—O3—C3	83.6 (3)	O2—C1—C2—Cl1	117.6 (3)
O15—Cr2—O4—C3	−39.5 (3)	O1—C1—C2—Cl1	−61.9 (3)
O11—Cr2—O4—C3	−133.4 (3)	Cr1—O3—C3—O4	28.9 (5)
O6—Cr2—O4—C3	55.3 (3)	Cr1—O3—C3—C4	−148.2 (2)
O1W—Cr2—O4—C3	141.2 (3)	Cr2—O4—C3—O3	1.6 (5)
O15—Cr1—O5—C5	−40.0 (3)	Cr2—O4—C3—C4	178.7 (2)
O1—Cr1—O5—C5	142.1 (3)	O3—C3—C4—Cl4	−161.7 (3)
O3—Cr1—O5—C5	53.7 (3)	O4—C3—C4—Cl4	20.9 (4)
O9—Cr1—O5—C5	−134.8 (3)	O3—C3—C4—Cl6	−38.2 (4)
O15—Cr2—O6—C5	−10.7 (3)	O4—C3—C4—Cl6	144.4 (3)
O13—Cr2—O6—C5	82.2 (3)	O3—C3—C4—Cl5	79.6 (3)
O4—Cr2—O6—C5	−104.8 (3)	O4—C3—C4—Cl5	−97.8 (3)
O1W—Cr2—O6—C5	168.9 (3)	Cr2—O6—C5—O5	34.3 (5)
O15—Cr1—O7—C7	−38.4 (3)	Cr2—O6—C5—C6	−144.0 (3)
O1—Cr1—O7—C7	139.6 (3)	Cr1—O5—C5—O6	−1.4 (5)
O3—Cr1—O7—C7	−131.9 (3)	Cr1—O5—C5—C6	176.9 (2)
O9—Cr1—O7—C7	56.5 (3)	O6—C5—C6—Cl9	−133.0 (3)
O15—Cr3—O8—C7	−5.6 (3)	O5—C5—C6—Cl9	48.5 (4)
O10—Cr3—O8—C7	−100.0 (3)	O6—C5—C6—Cl7	−9.9 (4)
O2W—Cr3—O8—C7	174.0 (3)	O5—C5—C6—Cl7	171.6 (3)
O12—Cr3—O8—C7	87.4 (3)	O6—C5—C6—Cl8	107.3 (3)
O15—Cr1—O9—C9	−7.1 (3)	O5—C5—C6—Cl8	−71.2 (3)
O1—Cr1—O9—C9	174.1 (3)	Cr1—O7—C7—O8	1.2 (5)
O5—Cr1—O9—C9	84.1 (3)	Cr1—O7—C7—C8	−179.7 (2)
O7—Cr1—O9—C9	−98.9 (3)	Cr3—O8—C7—O7	27.7 (5)
O15—Cr3—O10—C9	−40.5 (3)	Cr3—O8—C7—C8	−151.5 (2)
O8—Cr3—O10—C9	53.1 (3)	O7—C7—C8—Cl10	9.9 (4)
O14—Cr3—O10—C9	−134.1 (3)	O8—C7—C8—Cl10	−170.8 (2)
O2W—Cr3—O10—C9	139.2 (3)	O7—C7—C8—Cl12	132.2 (3)
O15—Cr2—O11—C11	−7.5 (3)	O8—C7—C8—Cl12	−48.5 (3)
O13—Cr2—O11—C11	−100.9 (3)	O7—C7—C8—Cl11	−109.4 (3)
O4—Cr2—O11—C11	86.1 (3)	O8—C7—C8—Cl11	69.9 (3)
O1W—Cr2—O11—C11	172.8 (3)	Cr1—O9—C9—O10	22.5 (5)
O15—Cr3—O12—C11	−35.3 (3)	Cr1—O9—C9—C10	−158.9 (2)
O8—Cr3—O12—C11	−128.6 (3)	Cr3—O10—C9—O9	7.7 (5)
O14—Cr3—O12—C11	58.9 (3)	Cr3—O10—C9—C10	−170.8 (2)
O2W—Cr3—O12—C11	145.0 (3)	O9—C9—C10—Cl4'	36.3 (7)
O15—Cr2—O13—C13	−34.4 (3)	O10—C9—C10—Cl4'	−145.0 (7)
O11—Cr2—O13—C13	59.8 (3)	O9—C9—C10—Cl13	−60.9 (4)
O6—Cr2—O13—C13	−128.7 (3)	O10—C9—C10—Cl13	117.9 (3)
O1W—Cr2—O13—C13	144.9 (3)	O9—C9—C10—Cl15	174.7 (4)
O15—Cr3—O14—C13	−2.0 (3)	O10—C9—C10—Cl15	−6.6 (5)
O10—Cr3—O14—C13	92.1 (3)	O9—C9—C10—Cl5'	166.5 (5)
O2W—Cr3—O14—C13	178.4 (3)	O10—C9—C10—Cl5'	−14.7 (6)
O12—Cr3—O14—C13	−95.5 (3)	O9—C9—C10—Cl14	56.3 (3)
O11—Cr2—O15—Cr3	−39.09 (14)	O10—C9—C10—Cl14	−124.9 (3)
O13—Cr2—O15—Cr3	53.69 (14)	O9—C9—C10—Cl3'	−80.5 (6)
O4—Cr2—O15—Cr3	−126.83 (13)	O10—C9—C10—Cl3'	98.2 (6)
O6—Cr2—O15—Cr3	138.90 (13)	Cr3—O12—C11—O11	−3.2 (5)
O11—Cr2—O15—Cr1	142.66 (13)	Cr3—O12—C11—C12	173.2 (2)
O13—Cr2—O15—Cr1	−124.55 (13)	Cr2—O11—C11—O12	31.1 (5)
O4—Cr2—O15—Cr1	54.92 (13)	Cr2—O11—C11—C12	−145.3 (2)
O6—Cr2—O15—Cr1	−39.35 (14)	O12—C11—C12—Cl17	23.3 (4)
O8—Cr3—O15—Cr2	140.83 (13)	O11—C11—C12—Cl17	−159.8 (2)
O14—Cr3—O15—Cr2	−40.99 (14)	O12—C11—C12—Cl18	147.1 (2)
O10—Cr3—O15—Cr2	−127.36 (13)	O11—C11—C12—Cl18	−36.0 (3)
O12—Cr3—O15—Cr2	53.92 (13)	O12—C11—C12—Cl16	−95.5 (3)
O8—Cr3—O15—Cr1	−40.94 (14)	O11—C11—C12—Cl16	81.4 (3)
O14—Cr3—O15—Cr1	137.24 (13)	Cr2—O13—C13—O14	−1.5 (5)
O10—Cr3—O15—Cr1	50.87 (14)	Cr2—O13—C13—C14	176.2 (2)
O12—Cr3—O15—Cr1	−127.86 (13)	Cr3—O14—C13—O13	25.1 (5)
O3—Cr1—O15—Cr2	−38.30 (14)	Cr3—O14—C13—C14	−152.6 (2)
O9—Cr1—O15—Cr2	143.55 (13)	O13—C13—C14—Cl19	131.3 (3)
O5—Cr1—O15—Cr2	56.58 (14)	O14—C13—C14—Cl19	−50.7 (3)
O7—Cr1—O15—Cr2	−125.67 (13)	O13—C13—C14—Cl20	10.2 (4)
O3—Cr1—O15—Cr3	143.45 (13)	O14—C13—C14—Cl20	−171.8 (2)
O9—Cr1—O15—Cr3	−34.69 (14)	O13—C13—C14—Cl21	−109.3 (3)
O5—Cr1—O15—Cr3	−121.66 (14)	O14—C13—C14—Cl21	68.7 (3)

### Hydrogen-bond geometry (Å, °)

**Table:**

*D*—H···*A*	*D*—H	H···*A*	*D*···*A*	*D*—H···*A*
O1W—H1W1···N1	0.84 (1)	1.97 (2)	2.791 (4)	166 (5)
O1W—H1W2···N2	0.85 (1)	1.97 (1)	2.810 (5)	173 (4)
O2W—H2W1···N3	0.85 (1)	1.88 (1)	2.719 (5)	171 (4)
O2W—H2W2···O2^i^	0.83 (1)	1.81 (1)	2.613 (3)	165 (4)

Symmetry codes: (i) −*x*+3/2, *y*−1/2, −*z*+3/2.
